# Clinical use of lentiviral vectors

**DOI:** 10.1038/s41375-018-0106-0

**Published:** 2018-03-22

**Authors:** Michael C. Milone, Una O’Doherty

**Affiliations:** 1 Department of Pathology and Laboratory Medicine, Perelman School of Medicine at the University of Pennsylvania, Philadelphia, PA USA; 2 Center for Cellular Immunotherapies, Perelman School of Medicine at the University of Pennsylvania, Philadelphia, PA USA

## Abstract

Viral vectors provide an efficient means for modification of eukaryotic cells, and their use is now commonplace in academic laboratories and industry for both research and clinical gene therapy applications. Lentiviral vectors, derived from the human immunodeficiency virus, have been extensively investigated and optimized over the past two decades. Third-generation, self-inactivating lentiviral vectors have recently been used in multiple clinical trials to introduce genes into hematopoietic stem cells to correct primary immunodeficiencies and hemoglobinopathies. These vectors have also been used to introduce genes into mature T cells to generate immunity to cancer through the delivery of chimeric antigen receptors (CARs) or cloned T-cell receptors. CAR T-cell therapies engineered using lentiviral vectors have demonstrated noteworthy clinical success in patients with B-cell malignancies leading to regulatory approval of the first genetically engineered cellular therapy using lentiviral vectors. In this review, we discuss several aspects of lentiviral vectors that will be of interest to clinicians, including an overview of lentiviral vector development, the current uses of viral vectors as therapy for primary immunodeficiencies and cancers, large-scale manufacturing of lentiviral vectors, and long-term follow-up of patients treated with gene therapy products.

## Introduction

The advent of molecular biology in the 1970s enabled the development of a variety of tools to manipulate nucleic acids and has transformed modern medicine. Molecular biology forms the foundation of numerous biotherapeutics, such as recombinant enzymes (e.g., factor IX in hemophilia), monoclonal antibodies (e.g., trastuzumab), and growth factors (e.g., erythropoietin). Gene therapy, which involves the delivery of DNA encoding a gene of interest into a cell with the intention of treating a disease, extends the power of molecular biology to potentially correct diseases such as those caused by genetic deficiencies (e.g., β-thalassemia due to a defect in the β-globin gene). Beyond correcting genetic deficiencies, gene therapy can also endow a cell or organism with capabilities not present in the natural state. Adoptive cellular therapy using genetically engineered T cells is one of the most notable examples. Using a synthetic gene, such as a chimeric antigen receptor (CAR) or cloned T-cell receptor (TCR), T cells can be endowed with the ability to recognize antigens that are not naturally recognized by their endogenous TCRs. This approach is capable of generating robust clinical responses even in patients with advanced B-cell malignancies that are highly refractory to other existing therapies [1].

Gene therapy via gammaretroviruses, lentiviruses, adenoviruses, and adeno-associated viruses is attractive because of the natural ability of viruses to enter into and deliver genetic material to cells [2]. Gammaretroviruses and lentiviruses are subtypes of retroviruses, which contain an RNA genome that is converted to DNA in the transduced cell by a virally encoded enzyme called reverse transcriptase. Although the use of gammaretroviral vectors is more common, especially in the research setting, the number of clinical trials using lentiviral vectors for gene therapy is increasing. This review discusses the development of lentiviral vectors and summarizes their current clinical investigation, particularly from a safety perspective.

## History of lentiviral vector development

### Lentivirus biology

The basic genes required for retroviral and lentiviral survival and function are the *gag*, *pol*, and *env* genes; *gag* encodes structural proteins, *pol* encodes enzymes required for reverse transcription and integration into the host cell genome, and *env* encodes the viral envelope glycoprotein [3]. All retroviruses have a similar life cycle. The life cycle begins when the mature virus gains entry to the cell either through direct membrane fusion or receptor-mediated endocytosis facilitated through the binding of glycoproteins within the envelope to their cognate receptors on the target cell’s surface. This initial fusion step is followed by a process of uncoating; at this stage, several viral proteins (including some Gag subunits) dissociate from the viral core. The viral RNA is converted to proviral double-stranded DNA through a complicated multistep process of reverse transcription. The proviral DNA then complexes with viral proteins to facilitate nuclear import and integration into the host genome. The process of integration is assisted by crucial viral proteins, such as integrase, and endogenous host cell transcription factors such as LEDGF [4]. The integrated proviral genome of wild-type lentivirus relies on host machinery to initiate and complete transcription and translation of viral proteins necessary to assemble infectious particles. The viral progeny then exit the cell through a process called budding, in which virions are released into the extracellular space from the plasma membrane unlike other viruses that often bud. Like many enveloped viruses, lentiviruses utilize the endosomal sorting complexes required for transport pathway to execute the complex budding process and release virions into the extracellular space [5]. During the budding process, endogenous membrane proteins present within the host cell can be incorporated into the envelope of the virion such as MHC molecules, which may affect the subsequent disposition of the liberated viral particles. The processes of viral egress and subsequent viral spreading are essential to the life cycle of wild-type lentivirus, but are not germane to the understanding of the fundamental features of replication-incompetent recombinant lentiviral vectors.

**Fig. 1:**
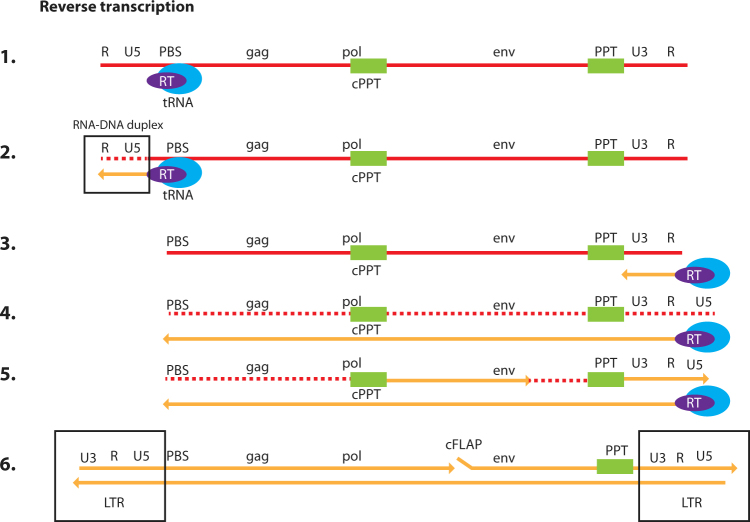
Steps of reverse transcription. This figure illustrates the steps involved in the conversion of the single-stranded RNA (ssRNA) genome of HIV into double-stranded DNA (dsDNA). RNA is shown in red and DNA as yellow [1]. The transfer RNA (tRNA) primer (blue ellipse) is base paired to the primer-binding site (PBS) [2]. Reverse transcription is initiated using the reverse transcriptase (RT; purple ellipse) enzyme, and minus-strand DNA synthesis starts from the tRNA primer, copying the U5 and R sequences at the 5′ end of the genome. At this stage, an RNA/DNA duplex is created, and ribonuclease (RNase) H activity of the RT enzyme degrades the viral RNA that has been copied (dotted red line) [3, 4]. The minus-strand DNA has been transferred, using the R sequence found at both ends of the viral RNA, to the 3′ end of the viral RNA, and minus-strand DNA synthesis continues. The HIV-1 genome has two RNase H-resistant polypurine tracts (PPTs) [5]. The two PPTs serve as primers for plus-strand DNA synthesis. One plus-strand is initiated at U3, and one is initiated at the central PPT (cPPT) [6]. Both the plus- and minus-strand DNAs are then elongated, finally resulting in a complete copy of viral RNA with additional sequences at the 5′ and 3′ ends such that viral DNA has an identical copy of U3RU5 at both ends. The plus-strand that was initiated at U3 displaces a segment of the plus-strand that was initiated from the cPPT, creating a small flap called the central flap (cFLAP). LTR long terminal repeat

Two unique steps of the retroviral life cycle, reverse transcription and integration, are integral to how lentiviral vectors function. Following uncoating, the remaining viral nucleic acid and protein complex is often referred to as the reverse transcription complex (RTC). This RTC is actively transported to the chromosomal DNA, where integration may occur [6–9]. During this migration, the viral RNA is converted within the RTC to double-stranded viral DNA by the reverse transcription process. This process begins when a transfer RNA binds to the primer-binding site at the 5′ end of the viral RNA genome (Fig. 1). As the negative-strand of the viral DNA is synthesized by the polymerase activity of the reverse transcriptase enzyme, the viral RNA is degraded by the RNAse H activity of this same enzyme. This process creates a short fragment of negative-sense, single-strand DNA, often referred to as strong-stop copy DNA (sscDNA). This sscDNA fragment is subsequently transferred to the 3′ end of the viral RNA to serve as a primer for the synthesis of the negative-strand viral DNA. In this process, the U3RU5 sequence of the long terminal repeat (LTR) that is essential for the viral life cycle is restored. Priming for the synthesis of the positive-strand viral DNA is accomplished through RNase H-resistant polypurine tracts, one close to the 3′ LTR and the other a central polypurine tract. In summary, the entire process uses multiple priming steps resulting in a complete DNA copy of the viral RNA. This viral DNA has an identical copy of the U3RU5 sequence within the LTR at both ends. As reverse transcription nears completion, the complex can be referred to as a pre-integration complex (PIC). Because there are potentially multiple priming events to synthesize the plus-strand, flaps of overlapping positive-strand DNA can form. It is thought that these flaps are likely repaired by host DNA repair enzymes after the process of integration [10, 11]. Although the central polypurine tracts (or the flaps of DNA they generate) are not essential for the process of reverse transcription, studies with viral vectors suggest that their presence enhances the potency of viral vectors. This may be due to the enhanced rate of second-strand synthesis, which may also protect the RTC from innate restriction factors, such as the apolipoprotein B mRNA editing catalytic polypeptide-like enzyme (APOBEC). However, it has also been suggested that this three-stranded DNA flap may also enhance nuclear import [12, 13]. Whether reverse transcription is completed in the cytoplasm or nucleus is unclear [10, 14].

The precise composition of the RTC may allow for more-effective reverse transcription. The capsid appears to protect the viral nucleic acid from innate sensors; simultaneously, it provides a structure that allows for more-effective reverse transcription with lower error rates. Reverse transcription is more accurate in cell lines than in a cell-free system, but the transcription rate is much slower (70 nt/min vs. 1000 nt/min). This rate is even slower still in primary cells, such as macrophages and resting T cells, which have lower nucleotide pools. The process of transporting the RTC from the cellular envelope to the chromatin of the cell is active, requiring energy [15], and it remains under-investigated; however, recent studies of the integration process have uncovered several host-virus interactions that may facilitate the process. One such model suggests that capsid protein has a fundamental role in the early part of the viral life cycle by utilizing cyclophilin A, cleavage and polyadenylation specificity factor 6 (CPSF6), Nup358, and TNPO3 to orchestrate a coordinated process of capsid uncoating, DNA synthesis, and integration that promotes evasion of the innate response and insertion into preferred areas of chromatin [16].

The steps of integration are (1) tethering (2), 3′ processing/cleavage of a precise number of terminal nucleotides (3), strand transfer, and (4) DNA repair. Important differences between gammaretroviruses (murine leukemia virus [MLV]) and lentiviruses (human immunodeficiency virus [HIV]) have consequences that should be considered when designing gene therapy vectors using retroviruses.

In the majority of retroviruses, the process of integration is not random. Each class of retrovirus has its characteristic preference [17, 18]. The gammaretrovirus MLV exhibits preferential insertion near transcriptional start sites, while the lentivirus HIV preferentially inserts within transcriptional units. The alpha retrovirus avian sarcoma leukosis virus has a milder preference for transcription units. The beta retrovirus mouse mammary tumor virus has no apparent preference. Gammaretroviruses, such as MLV, can gain access to the chromatin only after the nuclear envelope is dissolved. The tethering mechanism of MLV results in insertion sites within enhancers, and involves interactions between the p12 MLV protein with BRD2,3,4 (bromodomain and external domain proteins 2, 3, and 4) [19]. It also appears that DNA sequences that are highly bent due to wrapping around nucleosomes are favored [20].

Lentiviruses have additional selection criteria for integration that relate to their unique ability to translocate across the nuclear pore of an intact nuclear envelope. Gammaretroviruses must access the host genome during mitosis, when the nuclear envelope is disassembled, whereas lentiviruses can access chromosomes by active transport through the nuclear pore. Although the process of nuclear import is still largely not understood, viral proteins within the PIC, such as capsid, as well as elements of the reverse transcribed host genome are thought to be necessary for transport through the nuclear pore [19, 21]. Additionally, elegant studies by Yamashita et al. [21] demonstrated that the capsid protein of HIV but not MLV enables HIV’s translocation across the nuclear pore. In non-dividing cells, proximity to the nuclear pore also plays a role in integration site selection [22]. The heterochromatin tends to be associated with the nuclear envelope, while actively transcribed genes are closer to the nuclear pore [23]. Several host proteins within the nuclear pore (Nup153, Nup98, Nup358, CPSF6, and TPR) appear to play a role in HIV’s import. Host proteins LEDGF, BAF, and HMG are all associated with the PIC of HIV-1. LEDGF interacts with the PIC and with epigenetic (H3K36me3) marks, leading to the favored integration within transcription units. These unique properties of lentiviruses may be advantageous as certain gene therapy platforms target quiescent cell types, such as long-term hematopoietic stem cells (HSCs).

For many years, the consensus in the field was that HIV did not infect quiescent CD4 + T cells in the G0 stage of the life cycle but could infect activated CD4 + T cells [24, 25]. Around the same time, studies of patient cells demonstrated that quiescent CD4 + T cells contained HIV DNA [26]. Thus, it was hypothesized that the origin of infected resting CD4 + T cells was activated T cells that were infected and returned to a resting state. It was a bit puzzling that these quiescent CD4 + T cells, which expressed the receptors necessary for HIV fusion (CD4 and CXCR4, or CCR5), were resistant to HIV infection; nonetheless, the level of reverse transcripts were several logs lower in resting compared with activated CD4 + T cells when viral intermediates were measured shortly after inoculation (<24 h) [27]. Over time, it was recognized that the low level of nucleotides and high expression level of restriction factors in resting CD4 + T cells slowed the rate of reverse transcription [28, 29] and reduced the potential to infect resting CD4 + T cells directly but did not prevent the final outcome of reverse transcripts followed by integrated proviruses accumulating after 2 to 3 days in resting CD4 + T cells vs. 8 to 12 h in activated T cells [30–32].

An additional challenge for infection of resting cells by lentivectors was that quiescent CD4 + T cells fuse inefficiently with the commonly used G protein of the viral envelope vesicular stomatitis virus (VSV-G). VSV-G receptor-mediated endocytosis does not result in efficient infection of resting CD4 + T cells [33, 34] due to limited endocytosis [34, 35]. Treatment with several cytokines, such as interleukin 7, overcomes this limitation [36] and enhances cell survival, as G0 T cells are also prone to die soon after HIV infection [37].

It will be important to understand the effect of these restriction factors as these nuances to lentiviral biology will be important to consider during development of gene therapies based upon these vectors [29].

### Lentiviral vectors

First-generation lentiviral vectors contained a significant portion of the HIV genome, including the *gag* and *pol* genes, as well as several additional viral proteins [38]. The envelope protein of another virus, most commonly VSV-G, was included in the design of first-generation lentiviral vectors. VSV-G recognizes a ubiquitously expressed receptor that was most recently identified as the low-density lipoprotein (LDL) receptor [39, 40], allowing the lentiviral vector to transduce a wide range of cells [41]. The *VSV-G* gene was encoded on a separate plasmid from the other lentiviral genes. The lentiviral accessory genes *vif*, *vpr*, *vpu*, and *nef*, as well as the regulatory genes *tat* and *rev*, were included in first-generation lentiviral vectors. *vif*, *vpr*, *vpu*, and *nef* provide survival/fitness advantages for lentiviral replication in vivo, but they are not essential for the growth of the virus in vitro; *tat* and *rev* are required for viral replication.

Safer, second-generation lentiviral vectors lacking the accessory virulence factors *vif*, *vpr*, *vpu*, and *nef* were subsequently developed [38]. Removal of the accessory genes did not inhibit the transfer of genetic material to the host cell.

**Fig. 2:**
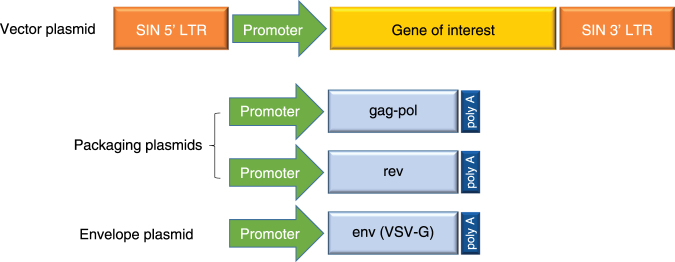
Third-generation lentiviral vector. Third-generation lentiviral vectors are composed of two separate packaging plasmids, one encoding *gag* and *pol* and another encoding *rev*. An additional plasmid encodes the envelope protein, derived from the VSV-G. The plasmid encoding the gene of interest contains lentiviral LTR sequences that have been altered to be self-inactivating (SIN) to prevent recombination. LTR long terminal repeat, VSV vesicular stomatitis virus

Third-generation lentiviral vectors further improved safety by splitting the viral genome into separate plasmids, making recombinant virus generation even more unlikely (Fig. 2) [42]. In the third-generation system, the *gag* and *pol* genes were encoded on a different plasmid from that of the *rev* or *env* genes, resulting in a vector made from three separate plasmids containing the necessary viral sequences for packaging. The *tat* gene was removed in third-generation lentiviral vectors due to its unnecessary function when a constitutively active promoter was engineered into the upstream LTRs of the construct containing the transgene. The introduction of deletions into the 3'LTR of the viral genome to create self-inactivating (SIN) lentiviral vectors disrupted the promoter/enhancer activity of the LTR, further improving safety [42].

The choice of internal promoters used in third-generation SIN lentiviral vectors is also important. Initial studies used the cytomegalovirus immediate early gene promoter, which showed robust expression in most cell lines that are actively dividing. However, promoters vary substantially in their activity in primary cells, such as CD34 + stem cells and T cells [43], with the cytomegalovirus promoter showing greater variation with T-cell activation than with constitutively active cellular promoters, such as elongation factor 1-α [44].

Although most current gene therapy approaches activate T cells to divide before transduction, the ability of HIV-1 to transduce non-dividing T cells in the G0 state [30] dependent upon the importation of the PIC across an intact nuclear envelope provides some hypothetical advantages for use in gene therapy. When cells are transduced in their resting, non-activated state, the cells may retain greater functional potential. For example, naive T cells have a very long intermitotic half-life, with estimates for humans of approximately 3.5 years [45, 46]. Similarly, slow cycling is also observed with HSCs, and long-term repopulating potential is inversely correlated with cycling [47]. By transducing non-dividing T or stem cells, the likelihood of persistence of the genetically modified cells may be increased.

Targeting non-dividing cells may also reduce oncogenic potential. Given that most retroviral vectors target transcriptional units, there is an increased chance of insertion into a transcriptional unit involved in cell division when transducing a dividing cell. This is suggested by clinical data in which the only cases of insertional mutagenesis were reported in genetically modified proliferative HSCs rather than quiescent cell types [48]. However, oncogenic potential is likely highly dependent on context, particularly preexisting genetic aberrations in treated patients [49]. Nonetheless, gammaretroviruses have demonstrated a considerable risk of leukemogenesis due to their integration patterns near proto-oncogenes. Lentiviral vectors can also insert near oncogenes, and tumor formation has been noted in at least one model that used a non-primate lentivirus-derived vector to transduce embryonic cells [50]. However, available clinical data suggest that newer generation vectors strongly reduce the risks of insertional mutagenesis as there are no reported cases to date of leukemogenesis in gene therapy trials that involve genetic modification of either HSCs or non-dividing T cells. Expansions of cells with a common integration site were observed in a gene therapy trial using lentiviral vectors for adrenoleukodystrophy [51]. Highly expanded CD4 + T-cell clones have also been observed in patients infected with HIV-1 [52, 53]. However, the clonal integration site expansions do not appear to be related to oncogenic selection [54].

Long-range chromatin interactions have also been shown to contribute to oncogenesis caused by retroviral vectors [55]. Montini et al. [56] demonstrated that by swapping regions of retroviral and lentiviral LTRs, lentiviral vectors can induce insertional mutagenesis when bearing the strong promoter/enhancer elements from retroviruses. SIN lentiviral vectors were shown to have a lower risk of insertional oncogenesis than gammaretroviral vectors in side-by-side comparisons of model systems [56, 57], and a mouse model of clinically relevant SIN lentiviral vectors showed that the genotoxic potential of lentiviral vectors can be diminished by the inclusion of an engineered chromatin insulator cassette [58]. However, as already discussed, clonal expansions can occur when lentiviral vectors integrate within known oncogenes; therefore, the risk of mutagenesis may be lower when using lentiviral vectors but is not eliminated [59]. An assay developed to test the oncogenic potential of gammaretroviral and lentiviral vectors may further improve safety when designing vectors for clinical use [60].

## Large-scale manufacturing of lentiviral vectors for clinical use

**Fig. 3:**
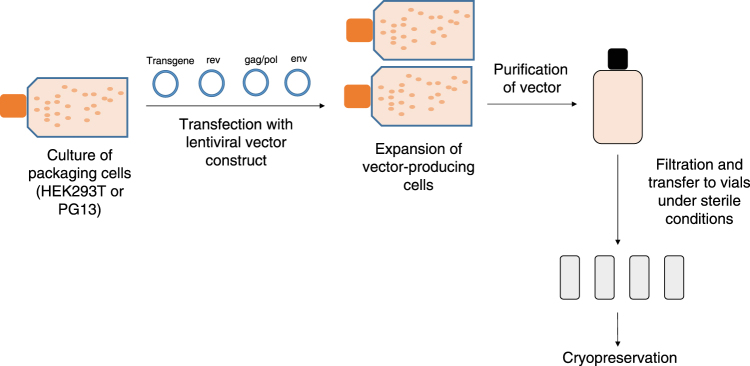
Overview of large-scale vector manufacturing. Manufacture of a lentiviral vector begins with the culture of a packaging cell line in a facility that uses Good Manufacturing Practices. The cells are transfected with the plasmids that make up the third-generation lentiviral vector, and the vector-producing cells are expanded in culture. The vector is purified from the cells and culture debris and filtered to ensure sterility, and individual aliquots are cryopreserved

The production of lentiviral vectors centers around the use of a cell line, typically referred to as a packaging cell, to produce the viral vector particles. Large-scale manufacturing of vectors begins with the growth of an adequate number of these packaging cells, such as derivatives of the HEK293T cell line (Fig. 3) [61]. An aliquot of packaging cells is expanded over several days in culture before the cells are then transiently transfected with plasmid DNA encoding the necessary proteins for lentiviral vector production. These core packaging plasmids include envelope protein (most commonly VSV-G), HIV-1 gag and pol gene products, and HIV accessory proteins, such as Rev. Co-transfection of these plasmids with the lentiviral vector genome containing the gene of interest provides all the components necessary for the cell line to produce functional vector particles. Over the course of several days, the packaging cells produce the lentiviral vector particles, which can be harvested from the culture medium. After flow filtration, the clarified vector is treated to remove contaminating DNA products, and viral products are purified using various methods such as gradient purification or chromatography. Following purification, the eluted fractions undergo a series of filtration steps for sterilization purposes and to remove any remaining cellular debris [62, 63]. The purity of the product is critical; debris from the packaging cells can easily be collected along with the vector product, and these impurities may cause inflammatory responses in vitro and in vivo [64]. Once generated, vector stocks have been reported to remain stable for up to 9 years when cryopreserved at −80 °C [61].

Generation of vectors for use in the clinical setting requires the use of current Good Manufacturing Practices (cGMP) to ensure production of high-quality vectors of verified identity, purity, and potency. The steps required for vector production are not complex; however, producing robust, cGMP-compliant production systems for lentiviral vectors has proven a bit more challenging than for producing retroviral vectors. Although lentiviral vector manufacturing has many parallels to gammaretroviral vector production, one of the key differences that significantly affects the manufacturing process consistency and increases costs of lentiviral vectors is the current lack of a packaging cell line with stable transfection of the core packaging plasmids. The need to transiently transfect three to four different plasmids with each vector manufacturing batch introduces opportunity for variation that is undesirable in a large-scale, cGMP manufacturing process. Attempts to produce stable lentiviral packaging cell lines comparable to those used in gammaretroviral vector production have largely failed in the past and is a major area of research in vector development. Recently, a method for generating clinical-grade stable packaging cell lines that continuously produce lentiviral vectors was reported [65]. This strategy used Cre recombinase-mediated insertion of a codon-optimized HIV-1 *gag*-*pol* construct into a constitutively expressed locus in 293FT cells. The remaining vector components were then transfected into a clone that expressed high levels of *gag*-*pol*. This approach solved a key issue with lentiviral vector production found in previous studies, which was that plasmid transfection of the *gag*-*pol* cassette did not induce continuous high-level expression. The availability of stable packaging cell lines could potentially expedite the development and manufacture of new lentiviral vector-based gene therapies as well as reduce the costs of this critical component of gene therapy. However, toxicity induced from expression of the other viral proteins used for packaging (i.e., Rev or VSV-G) also remains a challenge.

## Clinical use of lentiviral vectors

**Fig. 4:**
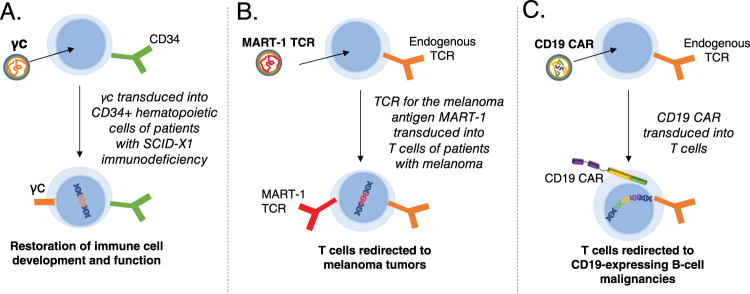
Key clinical uses of lentiviral vectors. **a** Correction of primary immunodeficiency. Using a viral vector to deliver the common gamma chain (γc) restores immune function in patients with SCID-X1. **b** Delivery of a tumor-specific T-cell receptor (TCR). A lentiviral vector can be used to introduce the MART-1 TCR, which recognizes a melanoma antigen, into a patient’s T cells ex vivo. The modified T cells, which now recognize melanoma cells, are administered to the patient as a cancer therapy. **c** Chimeric antigen receptor (CAR) T-cell therapy. A CAR engineered from three distinct domains (antigen recognition, co-stimulatory signaling, and T-cell signaling) can be introduced into T cells using a lentiviral vector. The cells expressing the modified receptor recognize the antigen of interest and harness the potent cytotoxic activity of T cells to attack tumor cells. Currently, most CAR T-cell therapies in clinical trials target the CD19 antigen, a protein expressed on B cells and B-cell malignancies. SCID severe combined immunodeficiency

Lentiviral and retroviral vectors are important technologies that are currently in development for a number of clinical applications requiring transfer of genetic material (Fig. 4). Lentiviral vectors have become particularly attractive for clinical applications due to their ability to more efficiently transduce non-proliferating or slowly proliferating cells, such as CD34 + stem cells. The first application of lentiviral vectors in the clinical setting used a conditionally replication-competent lentiviral vector encoding an anti-sense RNA targeting the HIV envelope gene. This vector was used to transduce mature peripheral blood T cells for the treatment of natural HIV infection. No adverse events attributable to the lentiviral vector were reported in this trial, which included up to 8 years of follow-up in some patients [66]. In addition, integration site analysis demonstrated preferential integration within transcribed genes as expected, with no significant change in the distribution of integration sites in the pre-infusion cellular product and engrafted T cells. Lentiviral vector-based gene transfer into CD34 + HSCs has subsequently been applied in the treatment of several genetic diseases, including β-thalassemia [67], X-linked adrenoleukodystrophy [51], metachromatic leukodystrophy [68, 69], and Wiskott-Aldrich Syndrome [70]. No adverse events related to the vector have been reported in these trials. In the initial study of HSCs transduced with β-globin in patients with β-thalassemia, one patient with β^E^/β^0^-thalassemia achieved independence from transfusion [67]. Interestingly, this response was associated with a relative increase in a dominant myeloid clone bearing a lentiviral vector insertion within the *HMGA2* gene locus. It is unknown whether the insertion within this dominant clone was merely a coincidence or selected based on enhanced proliferation resulting from dysregulation of the *HMGA2* gene. Several studies using lentiviral vectors to modify HSCs continue (Table 1), and longer follow-up will be necessary to fully establish the safety of lentiviral vectors in this therapeutic setting.

**Table 1:** Ongoing clinical trials using lentiviral vectors to modify hematopoietic stem cells

Condition	Phase	NCT number
Transfusion-dependent β-thalassemia	1/2	NCT02453477
	3	NCT02906202
Cerebral adrenoleukodystrophy	2/3	NCT01896102
Sickle cell disease	1	NCT02140554
	1	NCT02193191
Metachromatic leukodystrophy and adrenoleukodystrophy	1/2	NCT02559830
Wiskott-Aldrich syndrome	1/2	NCT01347346
	1/2	NCT01347242
	1/2	NCT02333760
X-SCID	1/2	NCT01306019
	1/2	NCT01512888
ADA-SCID	1/2	NCT02999984
	1/2	NCT01380990
Fanconi anemia	2	NCT02931071
X-linked chronic granulomatous disease	1/2	NCT02234934

*ADA* adenosine deaminase, *SCID* severe combined immunodeficiency

The safety of lentiviral vectors in ex vivo gene transfer into HSCs remains a somewhat open question; however, the field has now gained over 10 years of experience with lentiviral vectors for gene transfer into mature T cells. There have been several recent advances in cancer immunotherapy using genetically modified T cells. One approach involves the generation of cytotoxic T cells through the transduction of a tumor-specific TCR into a patient’s own T cells. Currently, ongoing phase 1 and phase 2 clinical trials are using autologous T cells that have been transduced to express the tumor antigens NY-ESO-1, MART-1, WT-1, and others [71]. In a trial using a lentiviral vector to transfer a TCR specific for a peptide that was shared by NY-ESO-1 and LAGE-1 as a therapy for patients with multiple myeloma, clinical responses were observed in 16 of 20 patients, with minimal safety concerns [72]. In addition, a lentiviral vector exhibited better transduction efficiency than a gammaretroviral vector in transducing T cells with a TCR targeting the Melan-A/MART-1 antigen [73], and a phase 2 trial of T cells transduced with a lentivirus to express MART-1 in patients with metastatic melanoma is ongoing. In some trials using TCR-modified cells, adverse effects caused by the transferred T cells have been observed; however, at present, there are no reports of adverse effects attributable to the use of lentiviral vectors in these studies.

In a similar approach to using TCRs to reprogram T cells, CARs targeting the B-cell marker CD19 introduced into T cells by both lentiviral and retroviral vectors have generated noteworthy clinical responses in patients with B-cell malignancies. Clinical trials using CD19-targeted CAR T-cell therapy for B-cell acute lymphoblastic leukemia have demonstrated high efficacy, with durable complete response rates in >60% of patients with relapsed or refractory disease [74, 75]. In addition, complete response rates of approximately 40 to 70% were observed in patients with non-Hodgkin lymphoma [76–78]. Several safety concerns with CAR T-cell therapies have been raised; however, as with TCRs, the adverse effects of CAR T-cell therapy appear to be mechanism based and not a result of using viral vectors [74].

One important on-target side effect of CD19 CAR T-cell therapy is B-cell aplasia, which is associated with long-term CAR T-cell persistence [74]. Because of the integrating nature of lentiviral vectors and the persistence of T-cell clones following adoptive transfer, lentiviral vector-modified T cells appear to be capable of persisting and inducing continued B-cell aplasia for >5 years following treatment. The half-life of these lentiviral vector-engineered T cells is currently unknown, but it may be lifelong. It is important to also recognize that individuals who have received lentiviral vector-based gene therapies may also exhibit positive testing on some HIV testing platforms, depending upon the vector construction and detection reagents used. Differentiating these from natural HIV-1 infection will, therefore, be important, and require more extensive testing.

Beyond the ex vivo modification of cells for adoptive transfer back into the patient, lentiviral vectors are also being applied directly in vivo for therapeutic purposes. A phase 1/2 study of a non-primate lentiviral vector based upon the equine infectious anemia virus (EIAV) expressing three genes involved in dopamine metabolism demonstrated the safety of local lentiviral gene delivery into the central nervous system with some evidence of clinical benefit [79]. In vivo gene delivery using a lentiviral vector has also been applied clinically to the eye [80]. These approaches face a number of hurdles including efficiency, need for tissue-restricted promoters, and immunogenicity. The latter is particularly important since immunogenicity can be related to both the delivered gene as well as components of the vector. As discussed earlier, the lentiviral vector envelope captures membrane proteins from the packaging cell lines during the budding process. Alloimmune reactivity towards HLA class I proteins carried within the vector envelope have been described and can limit vector survival. Gene editing of packaging cells to generate lines that lack HLA class I can enhance the stability of lentiviral vectors in serum [81]. Surface engineering in addition to vector genome engineering will likely be critical for successful application of these vectors in vivo.

## Regulatory aspects to the use of lentiviral vectors

In therapies using either gammaretroviral or lentiviral vectors, there is a theoretical potential for recombination events to occur during vector manufacturing that result in replication-competent retroviruses or lentiviruses (RCRs/RCLs) [82]. Incidence of insertional mutagenesis in patients could be increased if RCRs/RCLs are present in the vector, due to the potential for ongoing viral replication and insertion into the host DNA. Therefore, the US Food and Drug Administration (FDA), the European Medicines Agency, and most regulatory agencies require extensive testing for RCRs/RCLs in vector products as well as in patients [82, 83]. Testing for RCRs/RCLs is performed on the packaging cell lines, the purified vector product, and the genetically modified cellular therapy product before infusion into the patient. An assay that is commonly used to test the vector or cellular products is the S^+^L^−^ assay, which involves the incubation of the test sample with a cell line that enables viral replication [84]. Patients can be monitored for RCRs/RCLs through polymerase chain reaction or serological assays, and the FDA suggests that patients be monitored for recombinant virus every 3 months for the first year after receiving gene therapy. It has been suggested that the vigorous testing requirements for RCRs/RCLs should be revised due to the unlikely potential for generation of recombinant virus, the high cost of testing, and the labor involved [82]. The third-generation SIN lentiviral vector system makes the generation of RCLs very unlikely because the viral genome is split into separate plasmids.

Due to the theoretical possibility of RCRs/RCLs and secondary malignancies, health authorities in the United States and Europe require long-term follow-up for studies of cell and gene therapies that use viral vectors. The requirements for follow-up vary depending on the country; in the United States, monitoring of patients at least once a year for 15 years after receiving gene therapy is recommended. The long-term post-marketing surveillance required for the tisagenlecleucel (CTL019; Kymriah) that was recently approved by the FDA was in part driven by these theoretical concerns [85]. However, the optimal duration of patient follow-up depends on several factors, including vector persistence and transgene expression. In addition to monitoring patients for secondary malignancies and RCRs/RCLs, patients should be examined for new incidence or exacerbation of pre-existing neurological, rheumatologic, or autoimmune disorders. The FDA also requires that patients participating in clinical trials that use viral vectors receive information about their mechanism of action and the possible effects of DNA integration, including the risk of delayed malignancies [86]. To help establish follow-up procedures and collect data from patients treated with gene therapies, clinical trials for the follow-up of patients treated with specific gene therapy products are ongoing.

## Future directions and challenges

Several ongoing areas of research are aimed at further improving gene therapy with novel viral vector designs. Non-integrating lentiviral vectors (NILVs) have been investigated as a means of avoiding insertional mutagenesis. NILVs, which are deficient in the viral integrase protein, can transduce both dividing and non-dividing cells, and the viral genome remains present in the cell as an episome rather than integrating into the host genomic DNA [87]. It is expected from the non-integrating genome structure of NILVs that gene expression might be short-lived in dividing cells. Although this may be undesirable in some applications, this dilutional effect might be useful in some settings, such as CAR T cells, where long-term expression of the genetic payload may not be necessary.

NILVs have been used effectively as a vaccination strategy in pre-clinical models, resulting in cellular and humoral immunity as well as anti-tumor immunity [88]. NILVs can also be co-transduced into cells with zinc finger nucleases, which facilitate recombination of DNA encoded in the vector with a specific site in the host DNA [89]. In a pre-clinical study, zinc finger nucleases were used in T cells to replace the endogenous TCR with a tumor-specific TCR introduced by an NILV [90]. Replacing the endogenous TCR in this manner results in abrogation of off-target activity mediated by the endogenous TCR, thereby enhancing safety. To allow for long-term expression in dividing cells, a dual NILV vector system was developed to include the integrase of phage phiC31 [91].

Two types of cancer vaccines using lentiviral vectors have been investigated: dendritic cell vaccines and cancer cell vaccines. Dendritic cells loaded with peptide from a tumor antigen can be used as a vaccine against cancers expressing that antigen. One such dendritic cell vaccine, Sipuleucel-T, has been approved by the FDA as a prostate cancer therapy. Lentiviral vectors have been investigated as a method of expressing tumor antigens in or modifying co-stimulatory signals on dendritic cells to further enhance their efficacy [92, 93]. Lentiviral vectors have also been used to constitutively activate the MAP kinase pathway in dendritic cells, which resulted in enhanced anti-tumor responses in mice [94].

An alternative vaccine approach is the use of cancer cells, which already express the tumor antigens of interest, instead of dendritic cells, which must be loaded with peptide. A study of B-cell lymphoma cells lentivirally transduced with co-stimulatory proteins and interleukin-12 demonstrated that using these modified cells as a vaccine can result in enhanced immunogenicity to the parental lymphoma cell lines in murine models [95]. Lentiviral vectors have also been used to convert the K562 erythroleukemic cell line into artificial antigen-presenting cells that can be used for in vitro T-cell expansion and potentially in vivo vaccination similar to the previously-reported GVAX [96, 97]. Although more research is needed to determine clinical efficacy and safety of cancer vaccines developed using lentiviral vectors, these approaches may lead to novel therapeutic options for patients.

Lentiviral vectors have also been used to deliver components of gene editing, such as guide RNAs for the clustered regularly interspersed short palindromic repeats (CRISPR)-Cas9 system. The CRISPR-Cas9 system uses an RNA sequence to guide the Cas9 nuclease to create precise double-strand breaks, which can then be followed by homologous recombination to result in gene deletion or point mutations [98]. One study used a lentiviral CRISPR guide RNA library to introduce targeted mutations into embryonic stem cells, and subsequent identification of phenotypic mutants demonstrated the efficacy of this approach as an alternative to genetic screening using RNA interference [99]. There is some concern around the amplification of off-target effects with gene editing as a result of integrating viruses due to the persistent expression of the gene editing machinery. In order to overcome these potential limitations, Chen et al. [100] described an approach to limit the duration of editing by co-expressing a guide RNA that recognizes the CAS9 itself targeting the expression cassette to be destroyed. This system may be advantageous if it offers a shorter window of CRISPR expression over NILVs or AAV vectors. Short-term delivery via NILVs might also circumvent some of these challenges. Lentiviral vectors are also frequently used in the research setting to alter gene expression through the expression of short hairpin RNA or antisense RNA. Most of these approaches are still in early stages of development, and much further research is needed to determine whether lentiviral vectors can serve as a viable platform for delivering these gene editing tools for therapeutic purposes.

## Conclusion

Gene therapy using lentiviral vectors has emerged as a promising therapeutic option for several conditions. The first lentivirally transduced cellular therapy, tisagenlecleucel (CTL019, Kymriah), was approved in the United States in August of 2017 for the treatment of pediatric and young adult patients with acute lymphoblastic leukemia. Several additional cellular therapies based upon lentiviral vector-engineered cells are in late-phase development. Third-generation SIN lentiviral vectors, in particular, have demonstrated safety when used to transfer genes into both stem cells and T cells. Although there is a theoretical potential for insertional oncogenesis with lentiviral vectors, no cases have been reported with a natural HIV or gene therapy using lentiviral vectors. Continued follow-up of patients who have already received lentiviral vector-based gene therapies is still necessary to understand the long-term safety and efficacy of these vectors. Additional basic and clinical research to improve transduction efficiency and manufacturing are also still needed.
